# Lexical-semantic processing in preschoolers with Developmental Language Disorder: an eye tracking study

**DOI:** 10.3389/fpsyg.2024.1338517

**Published:** 2024-05-14

**Authors:** Ernesto Guerra, Carmen Julia Coloma, Andrea Helo

**Affiliations:** ^1^Centro de Investigación Avanzada en Educación, Instituto de Educación, Universidad de Chile, Santiago, Chile; ^2^Departamento de Fonoaudiología, Universidad de Chile, Santiago, Chile; ^3^Departamento de Neurociencias, Universidad de Chile, Santiago, Chile

**Keywords:** semantic processing, lexical retrieval, Developmental Language Disorder, semantic competition, eye tracking

## Abstract

This study examined lexical-semantic processing in children with Developmental Language Disorder (DLD) during visually situated comprehension of real-time spoken words. Existing evidence suggests that children with DLD may experience challenges in lexical access and retrieval, as well as greater lexical competition compared to their peers with Typical Development (TD). However, the specific nature of these difficulties remains unclear. Using eye-tracking methodology, the study investigated the real-time comprehension of semantic relationships in children with DLD and their age-matched peers. The results revealed that, for relatively frequent nouns, both groups demonstrated similar comprehension of semantic relationships. Both groups favored the semantic competitor when it appeared with an unrelated visual referent. In turn, when the semantic competitor appeared with the visual referent of the spoken word, both groups disregarded the competitor. This finding shows that, although children with DLD usually present a relatively impoverished vocabulary, frequent nouns may not pose greater difficulties for them. While the temporal course of preference for the competitor or the referent was similar between the two groups, numerical, though non-significant, differences in the extension of the clusters were observed. In summary, this research demonstrates that monolingual preschoolers with DLD exhibit similar lexical access to frequent words compared to their peers with TD. Future studies should investigate the performance of children with DLD on less frequent words to provide a comprehensive understanding of their lexical-semantic abilities.

## Introduction

Developmental Language Disorder (DLD) is a condition that impacts approximately 7% of the general population (Tomblin et al., 1997; Norbury et al., 2016). This condition shows a high degree of heritability, influenced by complex interactions between genetic and environmental factors (Mountford et al., 2022). Children with DLD experience linguistic difficulties that are not attributed to any known biomedical cause and have a significant impact on their daily functioning, often leading to a poor prognosis (Bishop et al., 2017). These linguistic challenges can be manifested in one or more language components, such as morphology, syntax, semantics, pragmatics, and narrative discourse (Bishop et al., 2017). The severity of these challenges varies widely among individuals (Ardanouy et al., 2023).

Despite the wide range of linguistic profiles of this condition, children with DLD often present deficits at the lexical-semantic level, as evidenced in several studies (Kail et al., 1984; McGregor et al., 2002; Andreu et al., 2012a). One of the earliest indicators of their lexical difficulties is a delay in early word acquisition (LaParo et al., 2004; Rice et al., 2008). Later, during both preschool and school years, children with Typical Development (TD) exhibit a faster lexical development compared to their peers with DLD (Dollaghan, 1987; Rice et al., 1990; Mainela-Arnold et al., 2010; Jones and Brandt, 2018; Dosi and Gavriilidou, 2020). This is probably explained by their difficulties in learning new words [see Jackson et al. (2021) and Marshall (2014), for reviews on the topic], which leads to smaller vocabulary size (i.e., number of words known) and lower vocabulary depth (i.e., how well they know those words, including its meanings and usage) compared to their age-matched peers with TD.

Besides the deficit in vocabulary size in children with DLD, they also exhibit difficulties in handling lexical-semantic information (McGregor and Appel, 2002; Sheng and McGregor, 2010; Drljan and Vuković, 2019). At the lexical level, children with DLD often require more time to accurately retrieve names in comparison to their peers when identifying objects (e.g., Leonard et al., 1983; Katz et al., 1992; Lahey and Edwards, 1996; see Haebig et al., 2019; Leonard et al., 2019 for more recent and related research). Furthermore, they tend to exhibit a higher frequency of naming errors in various contexts, including object and action naming, and story retelling when compared to their peers with TD (McGregor, 1997; Messer and Dockrell, 2006; Andreu et al., 2012b). For example, children with DLD display reduced naming accuracy for both nouns and verbs, with a notably higher rate of naming errors within the DLD population than observed in children with TD (Messer and Dockrell, 2006; Andreu et al., 2012b). These findings have led to the proposal of a lexical retrieval deficit in children with DLD.

At the semantic level, challenges for children with DLD are twofold: First, they show difficulties in forming semantic representations of concepts and, second, in establishing connections between words. Regarding semantic representations, research has consistently demonstrated that, unlike their peers with TD, children with DLD struggle to define both concrete (McGregor et al., 2002) and abstract concepts (Ponari et al., 2018). Concerning their lexical-semantic networks, children with DLD show sparser connections between words and lower levels of semantic organization (Sheng and McGregor, 2010; Drljan and Vuković, 2019). This has been demonstrated mainly through association tasks requiring verbal response. For instance, Sheng and McGregor (2010) showed that children with DLD produced fewer semantic responses, more clang associations (connections between words based on sound rather than meaning), and more errors compared to age and expressive vocabulary matched peers. More recently, using a similar word association task, Drljan and Vuković (2019) found that both preschool-aged (5–6 years) and school-aged (7–8 years) children with DLD display significantly fewer mature associations (paradigmatic and syntagmatic) and more immature ones (phonological, unrelated, echolalic, or omissions) than children with TD, showing a similar trajectory but a marked, delay especially in the early school years, in their semantic development. Similarly, Alt et al. (2004) examined the ability of children with DLD to establish new semantic links using a learning task involving artificial objects and actions. Their findings revealed that these children were less adept at forming new semantic connections compared to children with TD.

As shown earlier in this introduction, most of what we know about semantic representation in children with DLD comes from studies using production tasks, like naming, word definition tasks and semantic association tasks eliciting verbal responses. These studies infer lexical and semantic processing based on these verbal responses, focusing on the outcome rather than the underlying processes. In turn, our understanding of lexical retrieval and semantic processing in children with DLD during online processing remains limited. There are, nonetheless, a few studies using online tasks that have shown a deficit in lexical retrieval and semantic processing in children with DLD (e.g., McMurray et al., 2010; Andreu et al., 2012a; Helo et al., 2022). Andreu et al. (2012a), using an eye-tracking technique demonstrated a delay in the retrieval of lexical information in children with DLD compared with TD children. Specifically, children were slower than their age-matched peers in directing their gaze towards the visual referent of words (nouns or verbs) when they were named. Interestingly, no differences in the time course of gaze preference were observed when both groups were exposed to an attractive but unnamed object (red circle), discarding a general processing speed issue.

Lexical retrieval has also been studied using competition tasks. This involves presenting a word alongside an image of its referent as well as images of competing objects. In typical populations, hearing a word (e.g., “car”) generates a higher percentage of looks toward the referent but also, with a lower degree, to phonological (e.g., “carrot”) and semantically related objects (e.g., truck) compared to unrelated objects (e.g., glasses; Mirman and Magnuson, 2009). These tasks have also been used to study clinical populations. For instance, McMurray et al. (2010) investigated lexical retrieval by examining phonological competition in four groups of adolescents: one with DLD, another with cognitive impairments, a third group with both cognitive and language impairments, and a group of TD children. Participants were presented with spoken words alongside images of the word referent and two phonological competitors (cohort and rhyme competitors). The results indicated that adolescents with DLD and those with cognitive and language impairments exhibited a lower visual preference for the word’s referent. Also, they observed that children with language impairment directed more gazes toward cohort and rhyme competitors compared to their same-age peers without language difficulties. The authors interpreted these results as suggesting that the deficit in the retrieval process for children with language impairment, implies that the difficulties in retrieving word information may be related to an inability to inhibit phonological information.

In a more recent study, Helo et al. (2022) examined the processes of lexical retrieval and semantic connections between words in children with DLD by using a lexical competition task involving familiar words. To investigate this, they conducted four eye-tracking experiments in children with and without DLD assessing real-time competition when hearing a spoken word presented together with a visual (shape) and either a phonological or semantic competitor. The timing of the visual stimulus presentation before the spoken word varied (simultaneously or 3 s of previewing). The fourth experiment assessed exclusively semantic processing with 3 s of previewing of visual stimuli. The results of the three first experiments revealed that children with DLD experienced greater challenges than their age-matched peers in retrieving shape information during word recognition. Results from the fourth experiment (pure semantic task) indicated that both groups exhibited a preference for the semantic competitor over an unrelated object. However, when analyzing the timing and duration of this preference, distinct differences between groups emerged regarding the extent and strength of the semantic competition effect. The TD group showed a much larger semantic competition effect, suggesting a relatively weaker connection between words in children with DLD.

In summary, the reviewed evidence in children with DLD indicate that although there is extensive evidence of lexical-semantic difficulties among these children in tasks such as naming and semantic association tasks, our understanding of these challenges in real-time language processing is still limited. Similarly, evidence from online tasks shows that children with DLD face challenges in both lexical retrieval and establishing semantic connections when processing spoken words. Specifically, results from online word recognition tasks have shown that children with DLD experience a delay in matching spoken words with their referents, which may be indicative of slower lexical information retrieval (Andreu et al., 2012a). Furthermore, evidence from lexical competition tasks suggests that children with DLD encounter more pronounced interference from phonological competitors compared to their TD peers, suggesting difficulties in processing lexical-phonological information (see McMurray et al., 2010). Interestingly, the evidence also showed that while both children with DLD and TD children exhibit a semantic competition effect, this effect is shorter in children with DLD, even with familiar words. This finding suggests difficulties at the semantic level, specifically indicating weaker connections between words (Helo et al., 2022).

This study aims to extend the previous investigation in lexical retrieval and semantic connections in preschoolers with DLD by focusing on semantic competition, rather than phonological competition as in McMurray et al. (2010) and employing a more demanding task than that presented in Helo et al. (2022). By doing so, we aim to bridge the gap between knowledge in the online processing of lexical semantic information, using an online methodology to investigate how these children retrieve word information and process semantic relationships during real-time language comprehension. Concretely, we will explore whether children with DLD experience increased semantic competition when accessing a familiar word without prior time to explore the visual field.

## The present study

The primary objective of our study is to determine whether children with DLD present difficulties processing lexical-semantic information in a semantic competition task. To do so, an eye-tracking experiment was implemented in which monolingual preschool participants (one group with DLD and one with TD) heard a spoken word (e.g., “car”) that appeared simultaneously with a visual context, allowing only a limited time for exploring the visual scene. This experiment was carried out under two different conditions: (a) Target condition where the referent of the spoken word appeared together with a semantically related object (e.g., truck; semantic competitor) and (b) Competitor condition where a semantic competitor appeared with a distractor (e.g., glasses).

If children with DLD can retrieve semantic information as fast and accurately as children with TD, we should observe a preference for semantic-related objects in the visual context. Alternatively, if children with DLD in our sample have difficulties retrieving lexical information, we should observe a late and/or lower preference, compared to the control group, for the target and the semantic competitor both in those trials where the semantic competitor appears with the target and in those where it appears with the distractor.

## Methods

### Participants

The final sample consisted of 20 monolingual participants who were native Spanish speakers with DLD (6 girls and 14 boys, 5;9 years, min = 5;1, max = 6;6), and 20 monolingual participants who were native Spanish speakers with TD (6 girls and 14 boys, average age = 5;9 years, min = 5;1, max = 6;7). All children had normal hearing, as determined by audiometric screening, nonverbal cognitive abilities within the normal range, measured by Raven’s Colored Progressive Matrices (scores at or above the 25th percentile considered normal). Normal or corrected-to-normal vision, and no history of neurological or other conditions impacting language development, based on teacher reports. Children in the TD group met the same criteria, with the exception of the history of language difficulties.

### Selection of participants

Our study involved participants diagnosed with DLD, selected from students enrolled in integration programs for children with this disorder in their respective educational institutions. These students had received an initial diagnosis from speech therapists at their schools, following the criteria set by Chile’s Ministry of Education (Decree 170). This diagnostic process included the Test for the Evaluation of Phonological Simplification Processes (TEPROSIF-R; Pavez et al., 2008; Cronbach’s alpha = 0.90) and the Allen Toronto’s Exploratory Test of Spanish Grammar (Pavez, 2003), which measures grammatical skills through both expressive (Cronbach’s alpha = 0.77) and receptive (Cronbach’s alpha = 0.83) components. Typically, we observed diverse performance profiles among children with DLD. However, to be diagnosed with this disorder, encountering challenges in grammar is a critical criterion. Consequently, every child identified with DLD in the present study exhibited grammar skill deficits, falling below the expected level—specifically two standard deviations beneath the established Chilean norms—on either the expressive or receptive components of the Toronto Exploratory Test of Spanish Grammar. This assessment tool has been validated for its efficacy in distinguishing between children with DLD and those with TD in terms of grammatical abilities within a Chilean context, as shown by Pavez (2003). Besides, comprehensive medical, pedagogical, and psycho-pedagogical assessments were conducted to exclude any additional disorders affecting language development.

In addition, our research team independently evaluated each participant, focusing on language structure elements like grammar and lexical-semantic abilities. This assessment used the Spanish adaptation of the Clinical Evaluation of Language Fundamentals (CELF-4; Semel et al., 2003), a benchmark test for linguistic evaluation in children with DLD (Aguado et al., 2015). We administered four CELF-4 subtests: Formulated Sentences, Word Structure, Expressive Vocabulary, and Word Classes. The first two subtests assessed grammatical skills, while the latter two focused on lexical-semantic skills. Participants who scored below the 16th percentile on any subtest were identified as low performing. This evaluation confirmed that all participants with a prior DLD diagnosis had grammatical challenges, and some also had semantic difficulties. The control group, TD children, were matched by age and socio-economic status. Their evaluation also included the Expressive Vocabulary subtest of CELF-4.

All participants underwent additional testing: audiometry to exclude hearing issues (thresholds at or below 20 dB) and Raven’s Progressive Matrices to rule out cognitive impairments (no significant differences between groups were found in Raven’s test scores, see Table 1). Importantly, based on the reports from teachers at their respective schools, we confirmed that both the DLD and TD groups had no history of neurological or social problems.

**Table 1:** Participants’ means in Raven and CELF-4 subtests (with standard deviation, SD) for the corresponding pairwise contrasts (Welch two sample *t*-test, two-tailed).

CELF-subtests	DLD (Raw scores)	TD (Raw scores)	*t*-value	*p*-value
Formulated sentences subtest	1.94 (1.77 SD)	–	–	–
Word structure subtests	12.44 (4.07 SD)	–	–	–
CELF–expressive vocabulary	16.3 (4.34 SD)	–	–	–
Word classes subtests	11.79 (5.48 SD)	–	–	–
CELF–expressive vocabulary	16.3 (4.34 SD)	28.9 (8.66 SD)	−5.91	< 0.001
Raven scores	15.85 (4.13 SD)	17.65 (3.73 SD)	−1.41	0.17

### Apparatus

During the experiment, participants’ gaze was monitored through an EyeLink 1,000 Plus eye tracking system (SR Research, Ontario, Canada). The experiment was implemented with a sampling rate of 500 Hz in remote mode (instead of head-stabilized), as it is usual for studied with children. The images were presented on a high-precision 24-inch monitor (BenQ XL2430). Auditory stimuli were presented through headphones at a moderate volume.

### Materials and experimental design

For the experimental task, 20 auditory stimuli and 60 images of familiar objects belonging to 6 common categories (toys, fruits and vegetables, animals, furniture, means of transport, and school supplies) were used. The auditory stimuli consisted of Spanish words referring to 20 of these familiar objects, which were recorded by a female native Spanish speaker. Consequently, the images included 20 referents corresponding to the 20 auditory stimuli, 20 images representing semantic competitors of these referents, and finally, 20 images of objects unrelated to the objects referred to by the auditory stimuli. Table 2 presents the complete set of auditory and visual stimuli used in the experiment.

**Table 2:** Set of materials used in the experiment.

Spoken word and visual target	Semantic competitor	Distractor picture
auto (car)	camión (truck)	lentes (lenses)
betarraga (beet)	pepino (cucumber)	cuadro (picture)
buque (ship)	bote (boat)	gorro (cap)
camioneta (pickup truck)	taxi (cab)	anillo (ring)
chancho (pig)	vaca (cow)	monedero (purse)
coliflor (cauliflower)	cebolla (onion)	guitarra (guitar)
conejo (rabbit)	ratón (mouse)	estuche (case)
durazno (peach)	pera (pear)	violín (violin)
flecha (arrow)	espada (sword)	goma (rubber)
frutilla (strawberry)	melón (melon)	calcetín (sock)
león (lion)	hipopótamo (hippopotamus)	lápiz (pencil)
lobo (wolf)	canguro (kangaroo)	pantalón (pants)
martillo (hammer)	pala (shovel)	flauta (flute)
mesa (table)	cama (bed)	zapato (shoe)
monopatín (skateboard)	bicicleta (bicycle)	polera (shirt)
peluche (plush toy)	muñeca (doll)	bufanda (scarf)
refrigerador (refrigerator)	cocina (kitchen)	domino (domino)
regla (ruler)	destacador (highlighter)	camisa (shirt)
resbalín (slide)	columpio (swing)	piano (piano)
tenedor (fork)	cuchara (spoon)	reloj (watch)

The images of these objects were always presented in pairs, which gave rise to the two experimental conditions of the study depending on their combination. The Competitor-Target (CT) condition presented an object to which the spoken word referred more than its semantic competitor, while the Competitor-Distractor (CD) condition presented an object unrelated to the spoken word in addition to a semantic competitor for that word. Using a Latin square, these experimental conditions, plus the relative position of each object (left or right), were crossed in four experimental lists so that each participant was presented with the same number of repetitions in each experimental condition, with an equal number of referents on the left and right. At the same time, each spoken word appeared in each experimental condition in some list. In summary, the experimental design can be described as a one-factor design with two levels within-participant, within-item.

### Procedure

**Figure 1:**
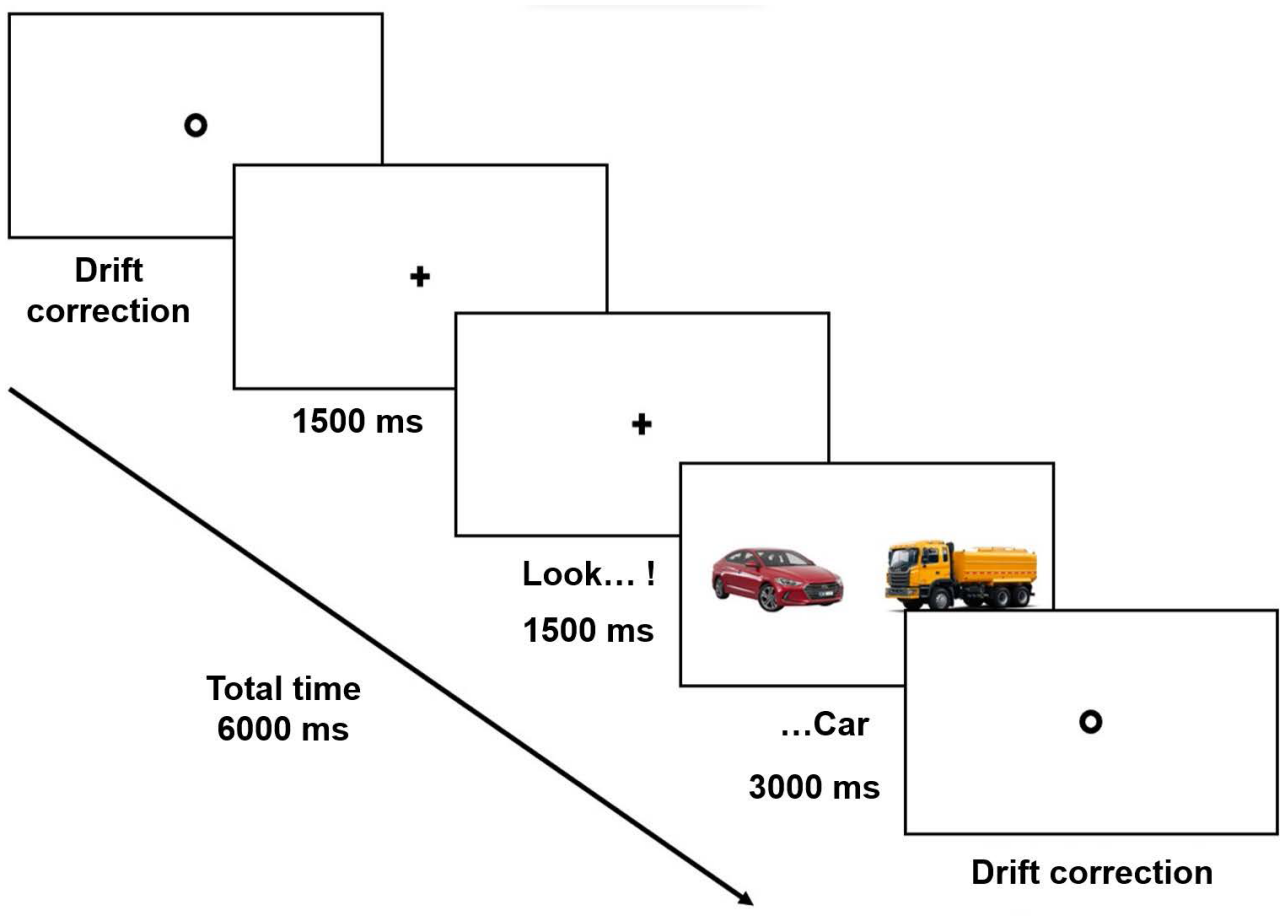
Schematic presentation of an experimental repetition in the CT condition.

All participants sat comfortably ≈60 cm from the computer screen in a room at their school. Before starting the experiment, 5 points of the eye-tracking system were calibrated and validated. Each child completed an experimental list with a total of 20 trials presented in random order. In each trial, a fixation point was presented in the center of the screen. Once the participant fixated on the central point, the experimenter manually activated the trial. Once the trial started, a cross appeared in the same place where the fixation point had been, and the imperative “Look!” was heard 1,500 ms after the start of the trial. The cross remained in the center for another 1,500 ms, after which the experimental materials (i.e., two images and one spoken word) were presented simultaneously. The images remained on the screen for another 3,000 ms. Once the repetition was completed, the fixation point that allowed the experimenter to start the next trial appeared again on the screen. Participants’ eye movements were recorded in each trial. There was no verbal communication with the children while the images were on the screen, but if necessary, the experimenter spoke to the children between repetitions to encourage them to continue. The experiment lasted approximately 5 min. Figure 1 shows a schematic representation of an experimental repetition in the CT condition.

### Data analysis

**Figure 2:**
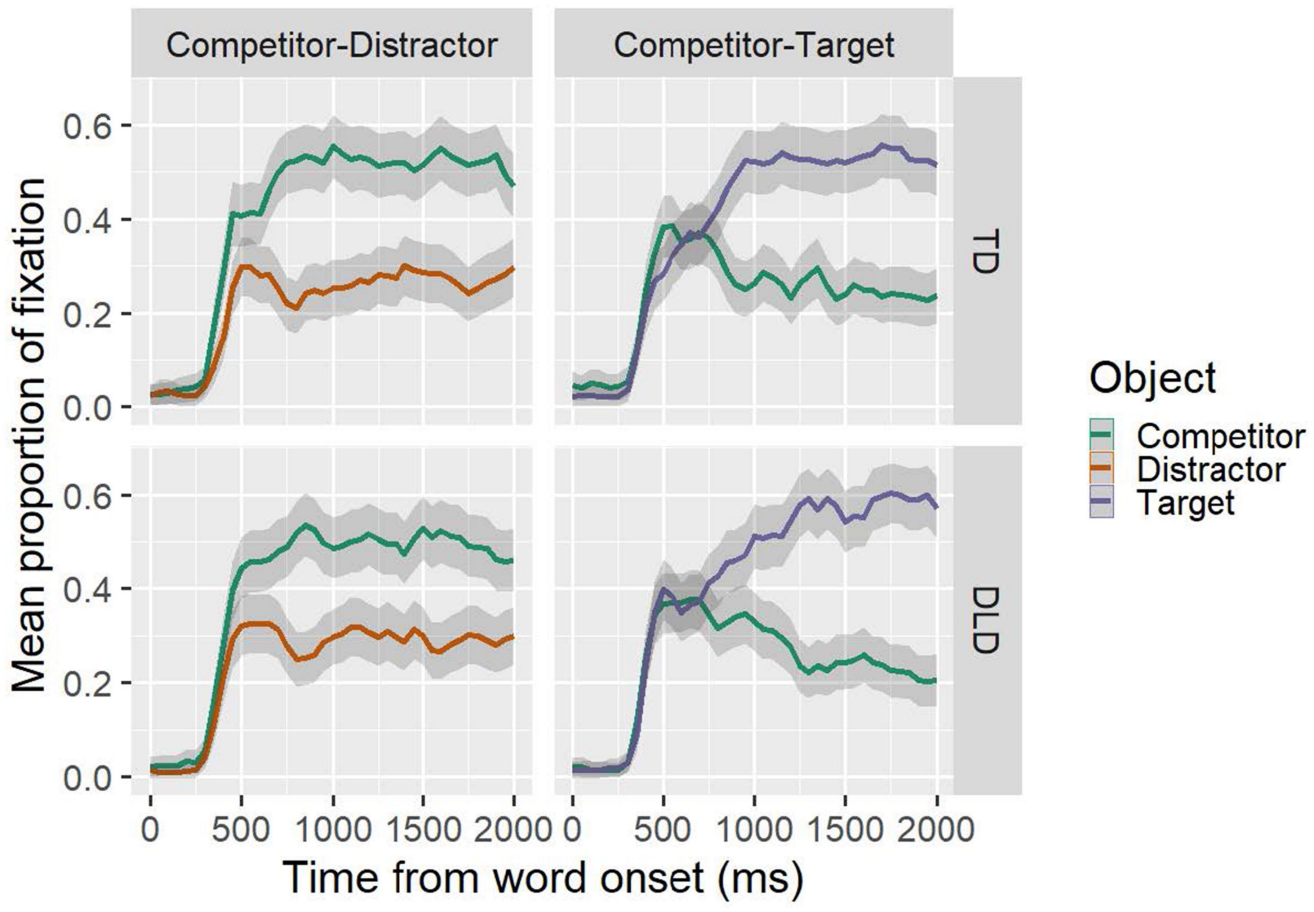
Temporal course of the average fixation proportion for each group in each experimental condition. The lines represent the different objects in the visual context, while the gray shaded area around each line represents the 95% confidence intervals (adjusted for within-participant designs).

Prior to statistical analysis, the Data Viewer software (SR Research) was used to create two areas of interest and extract a report with the duration and location of each fixation that occurred during each repetition. These areas of interest corresponded to the location and size of the objects presented in the visual context. The time window extended from the beginning of the critical spoken word to the end of the test. Subsequently, we used R Project software (R Core Team, 2018) to isolate each millisecond of the time window of interest and assign a value of 1 each time the participant’s gaze fell within one of the areas of interest and a value of 0 each time the participant’s gaze fell outside the areas of interest. Using the same software, these data were aggregated into 50 ms time windows for each participant, each item, and each area of interest. Finally, we calculated the average of the proportion of fixations to each area of interest, experimental condition, and group, along with the 95% confidence intervals (adjusted for within-participant designs, see Morey, 2008). Figure 2 provides an overview of the results, revealing the timing and magnitude of the participants’ visual preference for the images in the visual context.

To corroborate the confidence intervals, we used a nonparametric analysis based on random permutations of experimental condition labels in clusters (see Barr et al., 2014; Kronmüller et al., 2017; Chan et al., 2018; Kronmüller and Noveck, 2019; Barzy et al., 2020). Before implementing this analysis, we calculated our dependent variable defined as the logarithmic transformation of the ratio between visual preference for the semantic competitor and the accompanying image (i.e., the referent in the CT condition and the distractor in the CD condition), which we will call log-ratio (Arai et al., 2007). This variable provides a unique index of the difference between the proportion of fixations between the two objects present at the same time in the visual context. Positive values of the log-ratio reflect a preference for the semantic competitors, while negative values represent a preference for the other object (i.e., the referent or distractor depending on the experimental condition).

After these transformations, we carried out a cluster analysis. The first stage of analysis consisted of identifying the initial clusters from the contrast between the group of children with TD and the control group of DLD (for each experimental condition), as well as the contrast between the log-ratio and a distribution equal to zero (i.e., without object preference) for each group independently (see Barzy et al., 2020; Guerra et al., 2021; Christou et al., 2022a,b; Coloma et al., 2024). Using the lmerTest R package (Kuznetsova et al., 2017), we determined statistical significance for each 50 ms time interval by performing a mixed-effects linear regression on our dependent variable with group (i.e., TD, DLD, or zero) as a fixed effect and random intercept for participants and items. This was done for each time window and experimental condition separately. Subsequently, time windows composed of at least three consecutive 50 ms intervals showing statistical significance (*p* < 0.05) were aggregated.

The second stage consisted of constructing null hypothesis distributions through permutations. We created three null hypothesis distributions of *t*-values: one by randomly permuting the group labels (i.e., TD and DLD), and two others by randomly permuting the TD label with the ZERO label (from a distribution with no preference) and the DLD label with the ZERO label. All permutations were based on 2000 simulations in which each 50 ms time window was contrasted with randomly permuted labels. After obtaining the *t*-distributions, these values were aggregated by cluster and randomization, and then the absolute largest summed *t*-value was identified for each simulation, and finally these values were summed for each cluster.

The statistical significance of each cluster was determined by calculating the proportion between the sums of the highest *t*-values in the random distributions, which were greater than the sum of the *t*-values obtained for each cluster in our data. Following Chan et al. (2018), we considered proportions below 0.025 to be significant.

## Results

As can be seen in Figure 2, both groups preferred the semantic competitor in those repetitions in the CD condition and the visual referent in the repetitions in the CT condition. In this context, the cluster analysis did not detect significant differences between the groups (TD vs. DLD). In contrast, the clusters identified based on the contrast between each of the experimental groups and the zero distribution (TD vs. ZERO; DLD vs. ZERO) for each experimental condition appeared as significant.

Table 3 presents the experimental condition, the contrast, duration, observed sum of t values, and *p*-values for these clusters.

**Table 3:** Results of the cluster analysis.

Experimental condition	Contrast	Starts	Ends	*t* value	*p*-value
Competitor-Distractor (CD)	TD vs. ZERO	600	2000	105.58	<0.001
Competitor-Distractor (CD)	DLD vs. ZERO	500	2000	91.37	<0.001
Competitor-Target (CT)	TD vs. ZERO	850	2000	−104.10	<0.001
Competitor-Target (CT)	DLD vs. ZERO	1,000	2000	−109.96	<0.001

**Figure 3:**
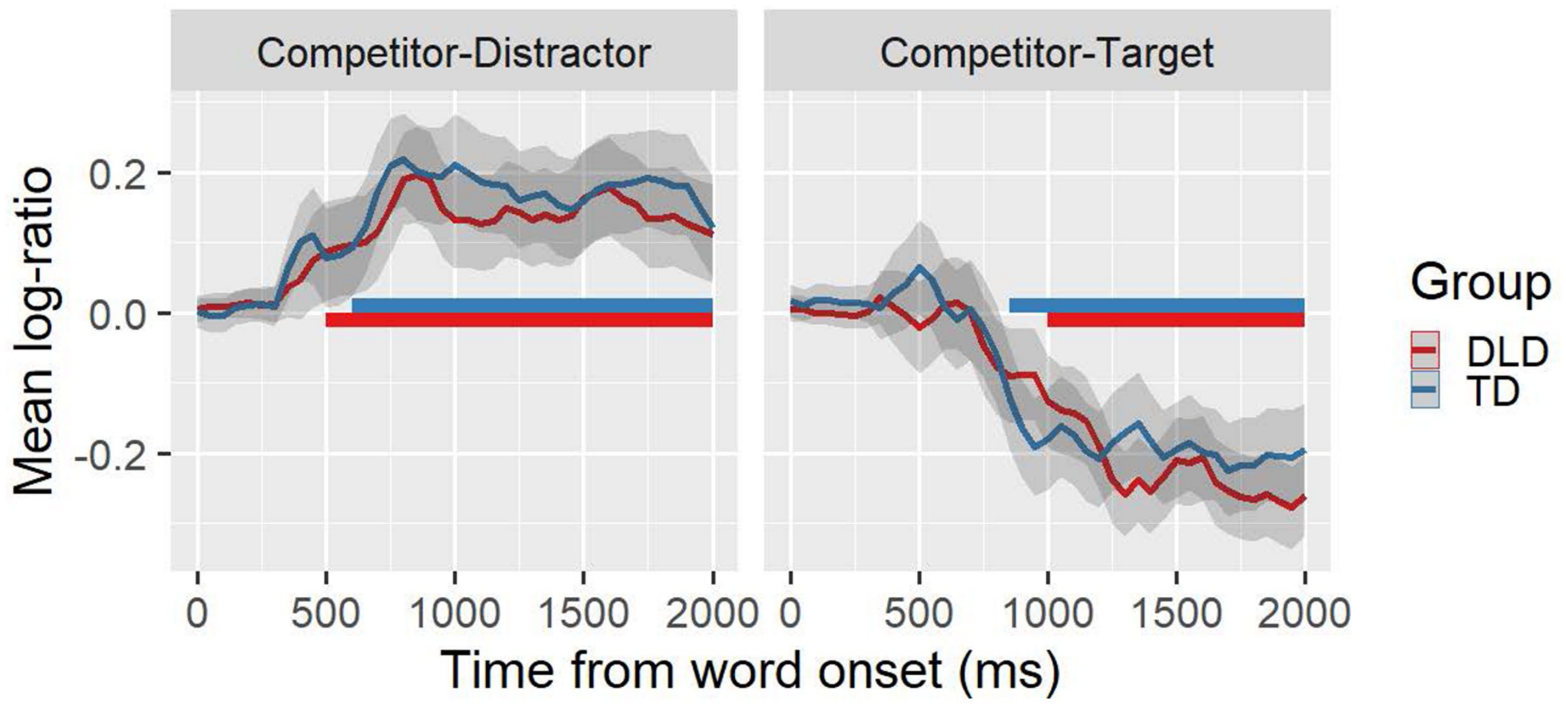
Temporal course of the average log-ratio (diverging lines) and extent of significant clusters (horizontal bars) for each group in each experimental condition. The lines of different colors represent the groups (TD, DLD), while the gray shaded area around each line represents the 95% confidence intervals (corrected for within-participant designs). The horizontal bars represent the extent of the clusters identified as significant.

As shown in Figure 3, participants from both groups quickly directed their gaze toward the semantic competitor shortly after the start of the spoken word in the CD condition. Specifically, 500 ms after the word onset for the TD participant group, and 600 ms for the DLD participant group. Similarly, both groups discarded the semantic competitor when presented in combination with the visual referent of the spoken word. Around 850 ms after the word onset, TD participants unequivocally directed their gaze towards the visual referent of the word, while the DLD participants preferred the visual referent after 1,000 ms from the word onset. Finally, as clearly shown in Figure 3, there were no significant differences between groups (TD vs. DLD) in the preference for the semantic competitor in any of the experimental conditions.

## Discussion

The aim of the present study was to investigate whether children with DLD experience difficulties in processing lexical-semantic information. To do so, children were engaged in a semantic competition task that involved the real-time comprehension of spoken words in a visually situated context while their eye movements were recorded. Spoken words were presented simultaneously as the visual objects appeared on the screen, providing children with limited time for visual exploration before processing the spoken word. Our results indicate a pattern of lexical access to frequent words and evidence a competition effect in preschoolers with DLD that is very similar to their peers with TD. These results contrast with existing evidence using production tasks (McGregor, 1997; McGregor et al., 2002; Messer and Dockrell, 2006; Ponari et al., 2018), where differences in lexical retrieval and semantic processing have been reported.

Specifically, our findings reveal that when interacting with relatively frequent words and objects, children with DLD shift their attention away from the semantic competitor and focus on the named object as TD children do, suggesting no significant difficulties in lexical retrieval processing. This observation contrasts with the findings of Andreu et al. (2012a), who reported a slower pace in matching spoken words with their visual referents in children with DLD compared with their age matched peers. However, it is important to notice that this difference was more pronounced for verbs than for nouns in their study. Given that our study involved very familiar nouns only, it is plausible that this familiarity facilitated word retrieval processing in children with DLD.

Furthermore, both groups demonstrated a preference for the semantic competitor while disregarding the unrelated object, exhibiting no statistically significant differences in the real-time activation of semantic relationships. These findings suggest that lexical-semantic processing of familiar words is preserved in children with DLD. However, these results contrast with the findings from Helo et al. (2022), who reported a shorter semantic competition effect in children with DLD compared to their age-matched peers, suggesting a weaker connection between semantically associated words in this group. In Helo et al. (2022), the visual objects were presented 3 seconds before the spoken word, whereas in the present study, the presentation of visual stimuli and the spoken word was simultaneous. This simultaneous presentation was designed to increase task difficulty compared to Helo et al. (2022), as it gave children no prior time to activate the semantic information of the objects on the screen. Despite this, the results show that children with DLD exhibit a pattern similar to TD children. Although this result might initially seem counterintuitive, previous research has demonstrated that differences between children with and without DLD are more apparent in easier tasks compared to more difficult ones (see Christou et al., 2022a). When presented with more challenging visual world tasks, both groups of children (TD and DLD) may require additional time or show increased uncertainty in identifying the relevant visual target, potentially masking group differences. Conversely, in easier visual world tasks, it is possible that only the DLD group falls behind.

It is important to notice that even though the temporal course of preference for the competitor and the referent were almost identical in both groups, minimal differences were observed (see Table 3). Children with DLD look slightly earlier at the semantic competitor (100 ms earlier) when it was presented with an unrelated object, and they look slightly later to the target (150 ms later) when it was presented with a semantic competitor. It is known that semantic competition is related to the activation and inhibition of lexical candidates (Rumelhart and McClelland, 1982). Thus, these two small differences could be linked to a greater lexical-semantic competition effect in participants with DLD similar to the greater phonological and semantic competition effect observed by McMurray et al. (2010) and Helo et al. (2022), respectively. Indeed, existing research has previously suggested that children with DLD are characterized by inhibitory inefficiency (Larson et al., 2020). A recent meta-analysis (Pauls and Archibald, 2016) demonstrated that children with DLD have inhibition difficulties, even though the severity of these difficulties depends on each child’s profile (Dispaldro et al., 2013). Such inhibitory deficit might allow irrelevant information to occupy working memory making processing less efficient and slower (see Marton et al., 2007). Previous work on young TD children (Marchman and Fernald, 2008) suggest that less efficient processing demand more extensive exposure to words to achieve comparable levels of representational depth, leading to slower vocabulary growth and weaker phonological and lexical relationships. Thus, these difficulties may be interconnected and potentially underlie the lower lexical skills observed in this population. However, since these differences were small and their direct contrast appeared not to be significant, further investigation is needed to corroborate this assumption.

Our study offers valuable insights into lexical-semantic competition in preschoolers with DLD but also presents several limitations. The relatively small, monolingual Spanish-speaking sample may limit the generalizability of our findings to other linguistic groups or bilingual populations. Additionally, focusing exclusively on high-frequency nouns might not fully represent the diverse lexical challenges faced by children with DLD, particularly with less frequent words or different parts of speech, such as verbs.

In sum, the results of this research show that lexical access to frequent words is as developed in monolingual preschoolers with DLD as in their TD peers, suggesting that semantic difficulties in this population are less severe in comprehension than production. Future research should confirm if this is the case for less frequent words. Regarding lexical competition, and without forgetting the absence of significant differences between the groups, children with DLD show a temporal course that suggests greater lexical-semantic competition, which could be related to the inhibition difficulties previously reported in this population. As a take-home message, our results contribute to a growing body of evidence suggesting that while children with DLD face challenges in linguistic abilities, they may be more resilient than previously understood.

## Data availability statement

The raw data supporting the conclusions of this article will be made available by the authors, without undue reservation.

## Ethics statement

The studies involving humans were approved by Comité de Ética, Facultad de Medicina. The studies were conducted in accordance with the local legislation and institutional requirements. Written informed consent for participation in this study was provided by the participants' legal guardians/next of kin.

## Author contributions

EG: Conceptualization, Data curation, Formal analysis, Funding acquisition, Methodology, Software, Visualization, Writing – original draft, Writing – review & editing. CC: Conceptualization, Data curation, Formal analysis, Funding acquisition, Methodology, Software, Visualization, Writing – original draft, Writing – review & editing. AH: Conceptualization, Data curation, Formal analysis, Funding acquisition, Methodology, Software, Visualization, Writing – original draft, Writing – review & editing.
